# Meat Exudate for Detection of African Swine Fever Virus Genomic Material and Anti-ASFV Antibodies

**DOI:** 10.3390/v13091744

**Published:** 2021-09-01

**Authors:** Chukwunonso Onyilagha, Mikyla Nash, Orlando Perez, Melissa Goolia, Alfonso Clavijo, Juergen A. Richt, Aruna Ambagala

**Affiliations:** 1 National Centre for Foreign Animal Disease, Canadian Food Inspection Agency, Winnipeg, MB R3E 3M4, Canada; chukwunonso.onyilagha@inspection.gc.ca (C.O.); nashm3@myumanitoba.ca (M.N.); orlando.perez@canada.ca (O.P.); Melissa.Goolia@inspection.gc.ca (M.G.); Alfonso.Clavijo@usda.gov (A.C.); 2 National Bio and Agro-Defense Facility, Agricultural Research Service, United States Department of Agriculture, Manhattan, KS 66506, USA; 3 Center of Excellence for Emerging and Zoonotic Animal Diseases, Department of Diagnostic Medicine/Pathobiology, College of Veterinary Medicine, Kansas State University, Manhattan, KS 66506, USA; jricht@vet.k-state.edu; 4 Department of Comparative Biology, Faculty of Veterinary Medicine, University of Calgary, Calgary, AB T2N 1N4, Canada

**Keywords:** African swine fever, meat exudate, ELISA, antibodies, real-time PCR

## Abstract

African swine fever (ASF) is one of the most important viral diseases of pigs caused by the ASF virus (ASFV). The virus is highly stable over a wide range of temperatures and pH and can survive in meat and meat products for several months, leading to long-distance transmission of ASF. Whole blood, serum, and organs from infected pigs are used routinely as approved sample types in the laboratory diagnosis of ASF. However, these sample types may not always be available. Here, we investigated meat exudate as an alternative sample type for the detection of ASFV-specific nucleic acids and antibodies. Pigs were infected with various ASFV strains: the highly virulent ASFV Malawi LIL 18/2 strain, the moderately-virulent ASFV Estonia 2014 strain, or the low-virulent ASFV OURT/88/3 strain. The animals were euthanized on different days post-infection (dpi), and meat exudates were collected and tested for the presence of ASFV-specific nucleic acids and antibodies. Animals infected with the ASFV Malawi LIL 18/2 developed severe clinical signs and succumbed to the infection within seven dpi, while pigs infected with ASFV Estonia 2014 also developed clinical signs but survived longer, with a few animals seroconverting before succumbing to the ASFV infection or being euthanized as they reached humane endpoints. Pigs infected with ASFV OURT/88/3 developed transient fever and seroconverted without mortality. ASFV genomic material was detected in meat exudate from pigs infected with ASFV Malawi LIL 18/2 and ASFV Estonia 2014 at the onset of viremia but at a lower amount when compared to the corresponding whole blood samples. Low levels of ASFV genomic material were detected in the whole blood of ASFV OURT/88/3-infected pigs, and no ASFV genomic material was detected in the meat exudate of these animals. Anti-ASFV antibodies were detected in the serum and meat exudate derived from ASFV OURT/88/3-infected pigs and in some of the samples derived from the ASFV Estonia 2014-infected pigs. These results indicate that ASFV genomic material and anti-ASFV antibodies can be detected in meat exudate, indicating that this sample can be used as an alternative sample type for ASF surveillance when routine sample types are unavailable or are not easily accessible.

## 1. Introduction 

African swine fever is a highly fatal viral disease of pigs [1]. It is a World Organization for Animal Health (OIE) notifiable disease, which significantly impacts the local and international trade of live swine and pork products. Until 1957, ASF was restricted to sub-Saharan Africa [2], where warthogs and bush pigs present asymptomatic infections, whereas domestic European pigs suffer severe clinical signs and high mortality. The first outbreak of ASF outside the African continent was reported in 1957 in Portugal, near Lisbon; the outbreak was caused by ASFV p72 genotype I virus-contaminated airline waste and was quickly eradicated. Three years later, the virus was re-introduced into Lisbon, Portugal [3], and spread to other European countries, the Dominican Republic, Haiti, and Brazil [4,5,6,7,8,9]. ASF was eradicated several decades later: in 1994 in Portugal and in 1995 in Spain; it is still present on the island of Sardinia [10]. The second epidemic of ASF outside Africa was reported in 2007 in Georgia, likely due to ASFV-contaminated pork or pork products (swill) obtained from ships anchored in the Black Sea port of Poti, which were accessed by free-ranging domestic pigs [11,12]. The outbreak was caused by an ASFV p72 genotype II virus that most likely originated in Eastern Africa [11,13,14,15]. The outbreak further spread to Europe and reached China in 2018 [16,17]. 

Currently, several countries in Southeast Asia, Europe, and Africa are facing the devastating economic impact of an ASF epidemic, where ongoing ASF outbreaks have caused the death of millions of pigs [18,19]. On 29 July 2021, ASF was reported in the Dominican Republic, 40 years after being eradicated from the Western Hemisphere [20]. The potential spread of ASF to North America is perceived as a serious risk for the pig industry, and the benefit of preventing ASF introduction into the U.S. alone was estimated to be worth approximately US $2.5 billion [21].

The clinical signs and gross lesions of ASF are not pathognomonic and can vary depending on the virulence of the virus [22], making laboratory confirmation essential. Highly virulent strains of ASFV cause an acute form of the disease characterized by high fever, depression, anorexia, hemorrhages in the skin, abortions, cyanosis, vomiting, diarrhea and death within 6–13 days, with mortality rates as high as 100% [23,24,25]. Moderately virulent strains produce milder clinical signs that can be present for a prolonged time period, with mortality rates generally ranging from 30–70% [26]. Low virulent ASF strains cause a chronic version of the disease, characterized by loss of weight, intermittent fever, respiratory signs, skin ulcers and arthritis.

The recommended samples for laboratory confirmation of ASF are blood or serum from live animals and organ samples (spleen, lymph nodes, bone marrow, lung, kidney, and tonsil) from dead animals [27]. The recommended samples, however, may not be convenient or economically feasible for ASF surveillance or simply not available in certain cases such as illegal pork imports or wild boar meat. Meat exudate, also called “meat juice”, “purge,” “weep”, or “drip”, is one of the alternative sample types proven viable for determining the health status of pig herds [28,29,30,31]; it can be used for the detection of a number of viral, protozoal, and bacterial pathogens of pigs. It is often mistaken for blood, but the red color of meat exudate is based on myoglobin, not hemoglobin. In addition to myoglobin, meat exudate contains water, glycolytic enzymes, amino acids, numerous water-soluble vitamins, and, depending on the anatomic location, traces of contaminating blood. Meat exudate is generated as a result of passive exudation, a complex phenomenon that is not fully understood [32,33]. 

The potential use of meat exudate for the detection of transboundary and endemic diseases has been explored previously. Genomic RNA of classical swine fever virus (CSFV) and foot and mouth disease virus (FMDV) have been successfully detected in meat exudate [34,35,36]. Meat exudate has also been used successfully employed for antibody-based serosurveillance of several other viral (influenza A virus, porcine circovirus 2, Aujeszky’s disease, porcine epidemic diarrhea virus, porcine reproductive and respiratory syndrome virus), bacterial (*Salmonella* spp., *Mycoplasma hyopneumoniae*, and *Yersinia enterocolitica*), and protozoal (*Trichinella *spp. and *Toxoplasma gondii*) pathogens of swine [28,29,30,31,37,38,39,40,41]. 

In this study, we evaluated the suitability of meat exudate as an alternative sample type for molecular and serological detection of ASF. Pigs were infected with high, moderately-high, and low virulent strains of ASFV, and the meat exudate was collected and tested for the presence of ASFV-specific genomic material and antibodies. 

## 2. Materials and Methods 

### 2.1. Ethics Statement

The animal experiments described here were conducted under the guidelines of the Canadian Council for Animal Care. The animal use document (AUD) C-1016-009 for this project was approved by the Animal Care Committee at the Canadian Science Centre for Human and Animal Health.

### 2.2. Pigs and Viruses 

Four to five weeks old Landrace-Large white cross-bred grower pigs used in this study were purchased from a local farm in Manitoba, Canada, and transported in a heated truck to the National Centre for Foreign Animal Disease (NCFAD) in Winnipeg, Canada (*n* = 65). Upon arrival, they were randomly assigned into groups and housed in biocontainment level 3 animal cubicles with ad libitum food and water supplies. The animals were monitored daily and given a 7 day of acclimatization period before they were used in the experiments. 

ASFV Malawi LIL 18/2 [42], ASFV Estonia 2014 [43], and ASFV OURT88/3 [44] isolates were used as the highly, moderately, and low virulent strains, respectively. All the viruses were propagated in primary porcine peripheral leukocyte culture (PPL) and titrated on primary porcine alveolar macrophages (PAM) following NCFAD standard procedures. Briefly, PPLs were isolated from freshly collected whole blood from naïve pigs and re-suspended in culture medium—RPMI-1640 (Mediatech, Manassas, VA, USA) supplemented with 1× Glutamax (2 mM), 5 mg/mL gentamicin (1% *v*/*v*), and 5% (*v*/*v*) γ-irradiated FBS (Thermo Scientific, Burlington, ON, Canada)—at 1 × 10^6^ WBC/mL; 1 mL/well of the suspension (plus 0.4% *v*/*v* RBC) was plated (24-well plate) and 0.2 mL of the previously titrated virus was used to inoculate the cells. The cultures were incubated for 7 days at 37 °C with 5% CO_2_, and cytopathic effect and hemadsorption were monitored. Further, tenfold dilutions of the virus-containing supernatants harvested from the cultures were used to inoculate PAM cells in culture medium—Minimum Essential Medium, Alpha (Mediatech, Manassas, VA, USA) containing 1% gentamicin (50 µg/mL), 1× Glutamax antibiotic solution, and 10% γ-irradiated FBS, followed by a 3-day incubation at 37 °C with 5% CO_2_. At the end of the incubation period, ASF Indirect Immunoperoxidase Assay (IPA) was performed, and the virus titers were calculated based on the positive wells.

### 2.3. Inoculation and Sample Collection

Fourteen pigs were infected with ASFV Malawi LIL 18/2 (Pigs 33–46), thirty-nine pigs with ASFV Estonia 2014 (pigs 1–39), and twelve pigs with ASFV OURT/88/3 (Pigs 63–74). Pigs were randomly assigned to pens (4–10 per pen) and infected with 1 × 10^5^ TCID_50_ ASFV Malawi LIL 18/2 or ASFV OURT/88/3 in 4 mL of culture media via the oronasal route (2 mL orally and 1 mL per nostril). For the ASFV Estonia 2014 study, pigs were infected with 2 × 10^5^ TCID_50_ ASFV Estonia 2014 (0.5 mL per nostril and 1 mL orally). On day 14, after the first ASFV OURT/88/3 infection, the pigs were re-infected with 10 times more ASFV OURT/88/3 (1 × 10^6^ TCID_50_). After infection, all animals were monitored twice daily, and the rectal temperatures were recorded. From all animals, whole blood and serum samples were collected on sampling days. Additional serum and whole blood samples were collected from all ASFV Malawi LIL 18/2 and ASFV OURT/88/3-infected pigs daily until the experiments were terminated. In the ASFV Malawi LIL 18/2 and ASFV Estonia 2014 experiments, two to four pigs were randomly selected (from 3 dpi) and euthanized daily for sample collection. In the ASFV OURT/88/3 experiment, two pigs were euthanized on 13 dpi, one pig on 19 dpi, three pigs on 21 dpi, three pigs on 25 dpi and three pigs on 26 dpi for sample collection. Following euthanasia, whole blood (in EDTA), serum, organ/tissue samples (spleen, bone marrow, and tonsils), and different muscle samples (only diaphragm samples in the ASFV Estonia2014 experiment) were collected. The muscle samples were cut into pieces of approximately 2 cm × 2 cm × 2 cm, put into clean, sealable plastic bags, and frozen at −20 °C until use. On the day before testing, the frozen meat samples were kept at 4 °C overnight to thaw. The meat samples were gently squeezed, and the exudate collected in the plastic bag was transferred to a clean tube with a pipette.

### 2.4. Quantitative Real-Time Polymerase Chain Reaction (qPCR) 

Total nucleic acid was extracted from the whole blood and meat exudate using the MagMAX™ Pathogen RNA/DNA Kit (Life Technologies, Burlington, ON, Canada) and the MagMAX Express-96 Magnetic Particle Processor (Life Technologies) following the manufacturer’s protocol. For the organ samples, nucleic acid extraction was performed on supernatant collected from 10% *w*/*v* suspensions in sterile PBS. The ASFV genomic material in the whole blood, organs, and meat exudate was quantified using a previously published TaqMan qPCR assay that specifically amplifies a conserved region of the p72 gene of the virus [45]. The ASFV qPCR was carried out using the TaqMan™ Fast Virus 1-Step master mix (Life Technologies) on the Applied Biosystems 7500 Real-Time PCR Instrument (Life Technologies) using the cycling conditions recommended for the master mix (50 °C for 10 min, 95 °C for 3 min followed by 40 cycles of 96 °C for 3 sec and 60 °C for 30 sec). The TaqMan RT-qPCR assay for beta-actin developed in-house was used to ensure valid nucleic acid extraction and the absence of PCR inhibitors in the samples [46]. Samples with Ct values of 35.99 and lower were considered positive, and values between 36 and 40 were considered suspicious. 

### 2.5. Enzyme-Linked Immunosorbent Assay (ELISA) 

Two commercially available ELISA kits were used in this study. Antibodies to ASFV in the serum samples were tested using INgezim PPA COMPAC blocking ELISA Kit (R.11.PPA.K.3, Ingenasa, Madrid, Spain), which uses a monoclonal antibody (MAb) specific to ASFV VP72 protein. This kit is not recommended for testing meat exudate due to the strong background observed. The I.D. Screen ASF Indirect ELISA kit (I.D.Vet, Grabels, France), a multi-antigen indirect ELISA kit for the detection of antibodies against ASFV P32, P62, and P72, was used to test for anti-ASFV antibodies in serum and meat exudate. Cut-off values for PPA COMPAC: <40%, negative; 40–50%, doubtful; ≥50%, positive. Cut-off value for I.D. Screen: <30%, negative; 30–40%, doubtful; ≥40%, positive. The units of measurements for the INgezim PPA COMPAC and I.D. Screen ASF Indirect ELISA kits are % inhibitions and S/P % ratios, respectively.

PPA COMPAC: Blocking % (x %) of a sample = ((NC−Sample OD)/(NC−PC)) × 100(1)

I.D. Screen: S/P % = (Net Sample OD/Net PC OD) × 100.(2)

NC, negative control; PC, positive control; OD, optical density. 

### 2.6. Statistics 

The Spearman’s correlation coefficient and *p*-value between whole blood and meat exudate as well as between serum and meat exudate were performed using GraphPad Prism version 8 for Windows (GraphPad Software, San Diego, CA, USA). *p*-values < 0.05 were considered statistically significant.

## 3. Results and Discussion

**Figure 1:**
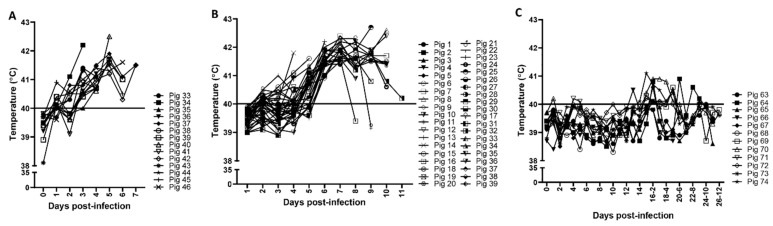
Rectal temperatures in pigs infected with ASFV Malawi LIL 18/2, ASFV Estonia 2014, or ASFV OURT/88/3. Groups of pigs (*n* = 12–39) were infected oronasally with 1 × 10^5^ TCID_50_ ASFV Malawi LIL 18/2 (**A**), 2 × 10^5^ TCID_50_ ASFV Estonia 2014 (**B**), or 1 × 10^5^ TCID_50_ ASFV OURT/88/3 (**C**), and on the indicated dpi, the rectal temperatures of the pigs were recorded and plotted. For the ASFV OURT 88/3 group, pigs were re-infected with a dose of 1 × 10^6^ TCID_50_ ASFV OURT 88/3 on 14 dpi. Cut-off = 40 °C.

Pigs infected with ASFV Malawi LIL 18/2 and ASFV Estonia 2014 developed fever (rectal temperatures above 40 °C for two consecutive days) starting from 3 dpi (Figure 1A,B). Additionally, these pigs displayed depression, diarrhea, nasal discharge, and vomiting. The pigs infected with low virulent ASFV OURT/88/3 did not develop any clinical signs including fever after the primary infection (Figure 1C); on 14 dpi, when they were re-infected with a 10 times higher dose of ASFV OURT/88/3, a few pigs transiently developed a mild fever but quickly recovered within a few days (Figure 1C); no other clinical signs were observed in the ASFV OURT/88/3-infected pigs. 

**Figure 2:**
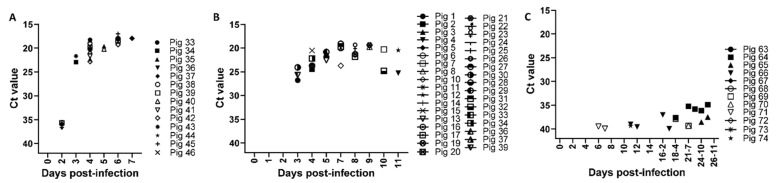
ASFV genomic material in whole blood samples from pigs infected with ASFV Malawi LIL 18/2, ASFV Estonia 2014, or ASFV OURT/88/3. Groups of pigs (*n* = 12–39) were infected oronasally with 1 × 10^5^ TCID_50_ ASFV Malawi LIL 18/2 (**A**), 2 × 10^5^ TCID_50_ ASFV Estonia 2014 (**B**), or 1 × 10^5^ TCID_50_ ASFV OURT/88/3 (**C**); on the indicated dpi, whole blood samples from the pigs were collected and assessed for ASFV genomic material using real-time PCR. For the ASFV OURT 88/3 group (**C**), pigs were re-infected with a dose of 1 × 10^6^ TCID_50_ ASFV OURT 88/3 on 14 dpi. Ct value of ≤ 35.99, positive. Dead pigs were excluded from the analysis.

**Figure 3:**
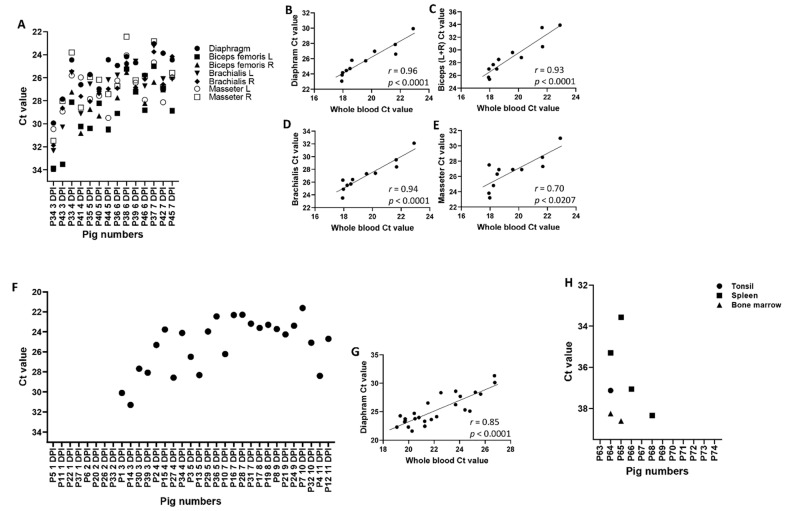
ASFV genomic material in the meat exudate (ASFV Malawi LIL 18/2 and ASFV Estonia 2014) and tissues (ASFV OURT/88/3) from infected pigs. Groups of pigs (*n* = 14–39) were infected oronasally with 1 × 10^5^ TCID_50_ ASFV Malawi LIL 18/2 (**A**) or 2 × 10^5^ TCID_50_ ASFV Estonia 2014 (**F**), and on indicated dpi, the respective muscles were collected from the pigs, and the extracted meat exudate was assessed for ASFV genomic material by real-time PCR (**A**,**F**). Spearman’s correlation was also performed between the whole blood and various meat exudate types in the ASFV Malawi LIL 18/2 (**B**–**E**) and ASFV Estonia 2014 (**G**)-infected groups of pigs. In another experiment, tissues from ASFV OURT/88/3-infected pigs (*n* = 12) were also assessed for ASFV genomic material by real-time PCR (**H**); for this group, pigs were first infected oronasally with 1 × 10^5^ TCID_50_ ASFV OURT 88/3 and re-infected with 1 × 10^6^ TCID_50_ ASFV OURT 88/3 on 14 dpi. Ct value of ≤35.99, positive; *p <* 0.05, significant. Dead pigs were excluded from the analysis.

ASFV-specific genomic DNA was detected starting from 2 and 3 dpi in whole blood samples collected from pigs infected with the highly virulent ASFV Malawi LIL 18/2 and the moderately virulent ASFV Estonia 2014, respectively (Figure 2A,B); most whole blood samples from low virulent ASFV OURT/88/3-infected pigs were negative, with a few samples (especially those collected after re-infection) being weak positive or suspicious (Ct values between 35.99 and 40) (Figure 2C). Interestingly, all the assessed meat exudate from the ASFV Malawi LIL 18/2 (Figure 3A) or ASFV Estonia 2014 (Figure 3F)-infected pigs from 3 dpi tested positive for the presence of ASFV genomic DNA. The Ct values obtained from meat exudate had strong positive correlations with those obtained from the corresponding blood samples from pigs infected with ASFV Malawi LIL 18/2 (Figure 3B–E) and ASFV Estonia 2014 (Figure 3G). However, the CT values in the whole blood samples were slightly lower (higher genome copy count) than those of the corresponding meat samples (Table 1A,B). ASFV genomic material was not detected in meat exudate collected from ASFV OURT/88/3-infected pigs. 

In the ASFV Estonia 2014 experiment, only the diaphragm samples were collected because the ASFV Malawi LIL 18/2 experiment established that meat juice from diaphragm samples contained comparable levels of ASFV genome copies to other muscle tissues. Importantly, diaphragm samples can easily be collected at slaughter without affecting the carcass quality, and several other studies have chosen meat exudate from diaphragm samples as a preferred alternative sample type for the detection of other swine pathogens [47,48,49,50].

Since only a few ASFV OURT/88/03-infected pigs showed low levels of ASFV genomic material in their blood and none of the meat exudate was ASFV qPCR positive, we wanted to determine the potential sites of ASFV replication in these pigs; therefore, spleen, tonsil, and bone marrow tissues were tested for the presence of ASFV genomic material by real-time PCR. Only two out of twelve pigs showed weak positive results in the spleen samples but not in the other tissue samples (tonsil and bone marrow) tested (Figure 3H). 

The meat exudate can also be used to detect antiviral antibodies [51]. To determine the potential of using meat exudate for detection of antibodies specific for ASFV, two commercial ASF serological kits, INgezim ASF PPA COMPAC and I.D. Screen ASF Indirect ELISA kits were used. While the I.D. Screen ASF Indirect ELISA kit is recommended for testing antibodies to ASFV in both serum and meat exudate samples, the INgezim ASF PPA COMPAC kit is recommended solely for testing serum samples. 

Serum samples collected from pigs infected with high-virulent ASFV Malawi LIL 18/2 had no detectable levels of anti-ASFV antibodies, as they all succumbed to infection by 7 dpi. Some pigs (pig #19, 25, 08, 21, 32, 4, and 12) infected with ASFV Estonia 2014 developed antibodies to ASFV in the serum samples as early as 8 dpi as detected by the INgezim ASF PPA COMPAC kit (Table 2A). However, only two pigs (pigs # 4 and #12) were positive for ASFV antibodies around 11 dpi by the I.D. Screen ASF Indirect ELISA kit (Table 2B). The difference in anti-ASFV antibody detection observed could be due to less sensitivity of the I.D. Screen ASF Indirect ELISA kit compared to that of the INgezim ASF PPA COMPAC kit. The serum samples were collected from these animals only once prior to euthanasia, and therefore the exact day these individual animals seroconverted could not be determined. 

Both ELISA assays were able to detect antibodies to ASFV in all pigs infected with ASFV OURT/88/3 (Table 2C,D). The INgezim ASF PPA COMPAC assay (Table 2C) detected ASFV-specific antibodies in the serum samples at earlier dpi (8 dpi) than the I.D. Screen ASF Indirect ELISA kit (10 dpi; Table 2D). However, both assays performed equally well when the antibody titers to ASFV increased during later stages of infection. 

**Figure 4:**
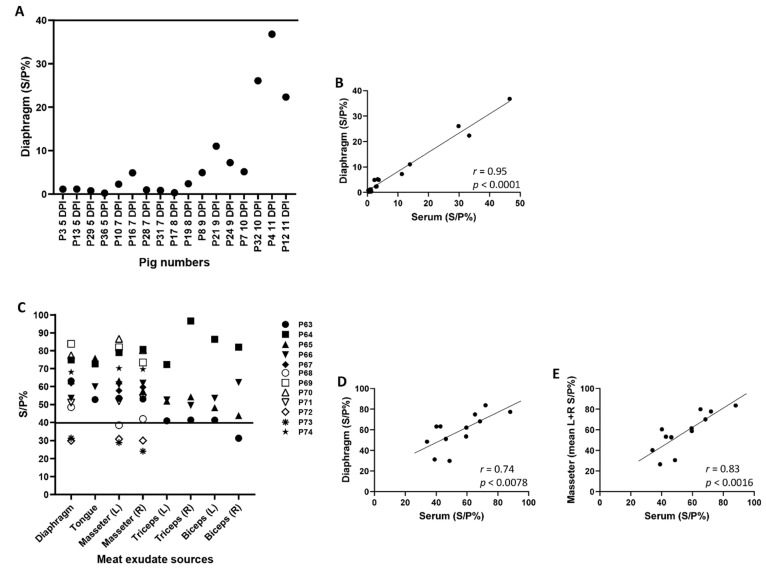
Anti-ASFV antibody levels in the meat exudate from ASFV Estonia 2014 or ASFV OURT/88/3-infected pigs. Groups of pigs (*n* = 12–39) were infected oronasally with 2 × 10^5^ TCID_50_ ASFV Estonia 2014 or 1 × 10^5^ TCID_50_ ASFV OURT/88/3. The ASFV OURT/88/3 group was re-infected with 1 × 10^6^ TCID_50_ ASFV OURT88/3 on 14 dpi. At the end of the experiment, the meat exudate from muscles of pigs infected with ASFV Estonia 2014 (**A**) and ASFV OURT88/3 (**C**) were assessed for antibodies to ASFV using the I.D. Screen ASF ELISA kit. Spearman’s correlation was also performed between the serum and meat exudate: diaphragm for ASFV Estonia 2014 group (**B**); diaphragm and masseter for ASFV OURT88/3 group (**D**,**E**). Cut-off value for ID Screen: <30%, negative; 30–40%, doubtful; ≥40%, positive. *p <* 0.05, significant. Values plotted are from 5 and 6 dpi for ASFV Estonia 2014 and ASFV OURT/88/3-infected groups of pigs, respectively.

Since none of the ASFV Malawi LIL 18/2 infected animals seroconverted, no anti-ASFV antibodies were detected in meat exudate samples. The meat exudate samples from the two seropositive ASF Estonia 2014-infected pigs (pigs #4 and #12) tested negative for antibodies to ASFV by I.D. Screen ASF Indirect ELISA kit (Figure 4A). However, the S/P% of diaphragm samples correlated positively with the corresponding serum samples (Figure 4B). 

Exudate collected from different meat samples (diaphragm, tongue, masseter, triceps and biceps) from ASFV OURT/88/3-infected pigs were tested for anti-ASFV antibodies using the ID Screen ASF Indirect ELISA kit. Meat exudate samples from all ASFV OURT/88/3-infected pigs that showed positive anti-ASFV antibodies in corresponding serum samples tested positive for anti-ASFV antibodies in the meat exudate samples, irrespective of the muscle type (Figure 4C and Table 3); a strong positive correlation between ASFV positivity in serum and meat exudate collected from the diaphragm (Figure 4D) and masseter (Figure 4E) muscles was detected. The number of meat exudate samples from the tongue, triceps, and biceps of the ASFV OURT/88/3-infected pigs was insufficient to perform correlation analysis. 

Although the levels of anti-ASFV antibodies in meat exudate samples were slightly lower than that in serum of ASFV Estonia 2014-infected pigs, the levels of anti-ASFV antibodies in the serum and meat exudate samples in ASFV OURT/88/3-infected pigs were comparable (Table 3). 

In situations where diaphragm samples are not available for testing, our study has provided evidence that other muscle samples could be used for detecting ASFV genomic material and anti-ASFV antibodies, as similar (and sometimes better) sensitivity levels can be achieved. However, variation in the results could arise depending on the quality of the sample.

One of the main methods of ASFV spread is through uncooked fresh and processed ASFV-contaminated pork products. Meat exudate is an easy obtainable sample from muscle tissues that can be collected at slaughterhouses, road kills, supermarkets, and the border from legally and illegally imported meat and meat products. Using ASFV strains with high, moderate, and low virulence, we demonstrated that meat exudate could be used as an alternative sample type to detect ASF-specific genomic material and antibodies. Based on these results, ASFV genomic DNA is readily detected in meat exudate, its presence coincides with viremia, and the amount correlates with the viral load in the blood. Therefore, when the testing of whole blood or organ samples is not possible, meat exudate could serve as an alternative sample type to identify ASFV-infected pigs that were viremic before they were slaughtered. This concept should hold true for wild boar meat (legal or illegal), illegal pork imports and pork products from illegally slaughtered ASFV-infected animals. The latter is a common practice by backyard, small-, and medium-size pig farmers in ASFV endemic countries. The study also confirmed that a low virulent ASFV strain causing chronic ASF does not result in reliably detectable viremia in the majority of infected animals; therefore meat exudate does not seem a suitable sample type for detection of ASFV genomic material in animals infected with low virulent ASFV strains. However, pigs infected with less virulent ASFV strains can be identified by the detection of ASFV-specific antibodies in meat exudate samples. The emergence of low virulent ASFV strains has been observed in ASF endemic areas in Europe (Estonia and Latvia) and Asia (China), as a result of natural evolution or due to the introduction of attenuated ASFV vaccine strains. Thus, failure to detect pigs infected with low virulent ASFV field strains is an important issue, especially in Asia. To overcome this problem, an abattoir-based meat exudate testing program for ASFV-specific antibodies could be a feasible solution. Unlike a serum-based surveillance program that requires skilled labor, handling of live animals, and use of needles and serum tubes, meat exudate-based surveillance requires fewer resources. Using easily accessible sample types such as diaphragm samples that can be easily obtained and scaled up, and will have no negative effect on carcass quality. As previously demonstrated for other pathogens, our data confirmed that diaphragm-based meat exudate samples could be used for detection of ASFV genome and anti-ASFV antibodies.

The I.D. Screen ASF Indirect ELISA kit used for the detection of anti-ASFV antibodies in meat juice samples appears to be less sensitive compared to the INgezim PPA COMPAC ELISA kit. However, the Ingezim PPA COMPAC ELISA is not suitable for meat exudate analysis. A more sensitive ASFV ELISA or point of care test that can detect anti-ASFV antibodies in meat exudate could overcome the failure to detect anti-ASFV antibodies in some meat exudate samples from pigs that showed weak positive serum antibody levels. In line with this, a recent report showed that a blocking ELISA based on ASFV P30 protein was highly sensitive and could be a great alternative for detecting antibodies against ASFV [52]. 

Conclusively, we have provided clear evidence that meat exudate is a suitable alternative sample type for detecting ASFV-specific nucleic acids and antibodies that could serve as a useful surveillance tool for ASF. Whole blood and organs, such as the spleen, remain the best samples for ASFV genome testing by real-time PCR, but meat exudate appears to be a feasible alternative sample type when these priority samples are not available. Commercial kits, evaluated/validated for use with meat exudate, can be used to detect antibodies to ASFV-proteins in meat exudate samples in order to obtain epidemiological information related to low and moderately virulent ASFV strains circulating in wild boars and domestic pigs, thereby facilitating ASF control and business continuity.

## Figures and Tables

**Table 1:** Side-by-side Ct values of the whole blood and meat exudate from pigs infected with ASFV Malawi LIL 18/2 or ASFV Estonia 2014.

**A**																									
**ASFV Malawi LIL 18/2: Whole Blood and Meat Exudate Ct Values**																									
				**P34** **3 DPI**		**P43** **3 DPI**		**P33** **4 DPI**		**P41** **4 DPI**		**P35** **5 DPI**		**P40** **5 DPI**		**P38** **6 DPI**		**P39** **6 DPI**		**P46** **6 DPI**		**P37** **7 DPI**		**P42** **7 DPI**	
**Whole blood**				22.9		21.7		18.2		21.7		19.6		20.2		18.0		18.5		18.6		17.9		17.9	
**Diaphragm**				29.9		27.9		24.5		26.6		25.7		27.0		24.2		24.7		25.8		23.1		23.9	
**Biceps (L)**				33.9		33.5		28.1		30.2		30.4		28.2		25.3		27.2		28.8		25.0		26.9	
**Biceps (R)**				34.0		33.5		27.2		30.8		28.7		29.3		25.5		26.8		28.2		26.4		27.0	
**Brachialis (L)**				32.3		30.3		25.6		29.1		26.5		27.4		25.0		24.6		26.7		23.2		26.1	
**Brachialis (R)**				31.9		28.6		25.5		27.6		28.1		27.4		24.7		26.9		26.1		23.8		26.6	
**Masseter (L)**				30.5		28.9		25.8		26.0		27.8		27.6		24.1		26.4		27.9		24.7		28.1	
**Masseter (R)**				31.5		28.0		23.8		28.6		25.9		26.2		22.4		26.2		25.8		22.8		26.8	
**B**																									
**ASFV Estonia: Whole Blood and Diaphragm Ct Values**																									
	**P1 3 DPI**	**P14 3 DPI**	**P30 3 DPI**	**P39 3 DPI**	**P2 4 DPI**	**P15 4 DPI**	**P27 4 DPI**	**P34 4 DPI**	**P3 5 DPI**	**P13 5 DPI**	**P29 5 DPI**	**P36 5 DPI**	**P10 7 DPI**	**P16 7 DPI**	**P28 7 DPI**	**P31 7 DPI**	**P17 8 DPI**	**P19 8 DPI**	**P8 9 DPI**	**P21 9 DPI**	**P24 9 DPI**	**P7 10 DPI**	**P32 10 DPI**	**P4 11 DPI**	**P12 11 DPI**
**Whole blood**	26.8	26.7	24.1	25.7	24.4	20.5	23.7	22.2	21.5	22.5	20.8	21.3	23.7	20.0	19.1	19.7	21.8	21.3	19.7	19.3	19.7	20.3	24.8	25.2	20.4
**Diaphragm**	30.1	31.3	27.7	28.1	25.3	23.8	28.6	24.1	26.5	28.3	24.0	22.5	26.2	22.3	22.3	23.2	23.6	23.3	23.7	24.3	23.4	21.6	25.1	28.4	24.7

Groups of pigs (*n* = 14–39) were infected oronasally with 1 × 10^5^ TCID_50_ ASFV Malawi LIL 18/2 (**A**) or 2 × 10^5^ TCID_50_ ASFV Estonia 2014 (**B**), and on the final dpi of each pig, the muscles were collected, and the extracted meat exudate was assessed for ASFV genomic material by real-time PCR. The results are presented together with those of the whole blood samples of the same dpi. Dead pigs were excluded from the analysis.

**Table 2:** Anti-ASFV antibody levels in the serum samples from ASFV Estonia 2014 and ASFV OURT/88/3-infected pigs.

**A**																			
	**P3** **5 DPI**	**P13** **5 DPI**	**P29** **5 DPI**	**P36** **5 DPI**	**P10** **7 DPI**	**P16** **7 DPI**	**P28** **7 DPI**	**P31** **7 DPI**	**P17** **8 DPI**	**P19** **8 DPI**	**P23** **8 DPI**	**P25** **8 DPI**	**P8** **9 DPI**	**P21** **9 DPI**	**P24** **9 DPI**	**P7** **10 DPI**	**P32** **10 DPI**	**P4** **11 DPI**	**P12** **11 DPI**
**Serum**	13.0	16.2	15.8	22.1	33.5	14.6	25.8	18.8	21.7	56.5	38.9	40.8	45.1	44.9	36.7	37.5	84.2	83.0	46.0
**B**																			
	**P3** **5 DPI**	**P13** **5 DPI**	**P29** **5 DPI**	**P36** **5 DPI**	**P10** **7 DPI**	**P16** **7 DPI**	**P28** **7 DPI**	**P31** **7 DPI**	**P17** **8 DPI**	**P19** **8 DPI**	**P23** **8 DPI**	**P25** **8 DPI**	**P8** **9 DPI**	**P21** **9 DPI**	**P24** **9 DPI**	**P7** **10 DPI**	**P32** **10 DPI**	**P4** **11 DPI**	**P12** **11 DPI**
**Serum**	0.9	1.1	0.3	0.5	2.8	2.3	0.8	0.0	1.3	3.0	1.9	2.5	3.7	13.9	11.3	3.4	29.8	46.6	33.3
**C**																			
**Pig number**	**DPI 0**	**6**	**7**	**8**	**9**	**10**	**11**	**12**	**13**	**14**	**16-2**	**17-3**	**18-4**	**20-6**	**21-7**	**23-9**	**24-10**	**25-11**	**26-12**
63	5.0	9.9	8.0	25.9	37.8	38.2	33.4	41.6	39.5	33.9	35.2	39.9	42.1	58.7	61.7	73.9	70.5	71.2	
64	10.5	10.4	13.5	24.8	26.3	32.9	19.6	26.1	19.9	15.9	13.6	15.1	17.3	73.6	74.7	75.4	69.1	70.1	
65	14.5	17.2	1.9	16.8	23.6	21.4	10.7	16.6	11.5	8.5	8.4	8.2	14.0	40.5	48.8	69.9	62.3	58.0	
66	5.6	9.1	3.8	24.7	37.6	37.9	34.4	42.4	47.3	43.0	50.3	58.1	64.5						
67	9.8	15.2	3.1	14.7	13.1	17.8	6.5	10.4	10.2	5.7	10.4	7.5	12.6	54.8	66.5				
68	9.5	13.8	17.2	33.8	48.8	51.2	52.0	54.6	63.5										
69	13.8	18.6	5.3	24.8	22.5	30.1	12.0	24.6	29.7	15.4	13.4	11.9	12.4	56.7	57.5	81.9	70.2	75.9	70.6
70	8.6	14.7	4.1	19.2	17.4	21.4	14.4	22.2	28.8	12.9	12.1	10.7	15.1	40.1	56.1	44.0	43.7	60.7	60.2
71	10.3	11.1	17.6	43.6	49.5	48.7	50.6	51.8	63.4										
72	11.3	15.6	8.0	22.6	29.7	32.3	25.0	28.8	34.7	20.9	21.2	22.0	22.2	46.4	63.8				
73	11.9	11.5	7.5	25.0	27.2	37.3	46.8	44.4	49.5	35.9	25.1	35.3	44.8	59.6	63.3	65.0	62.2	64.9	62.8
74	13.0	11.4	3.2	19.5	19.7	18.6	19.2	11.6	26.3	16.1	16.2	28.3	39.6	83.4	85.1				
**D**																			
**Pig number**	**DPI 0**	**6**	**7**	**8**	**9**	**10**	**11**	**12**	**13**	**14**	**16-2**	**17-3**	**18-4**	**20-6**	**21-7**	**23-9**	**24-10**	**25-11**	**26-12**
63	1.0	0.3	1.0	10.4	21.1	27.4	34.1	34.5	39.9	41.4	42.0	44.5	50.1	40.7	50.6	43.7	48.7	42.8	
64	0.7	0.4	0.5	0.2	1.0	1.2	1.6	1.5	1.5	2.4	9.8	16.1	27.8	69.6	81.4	78.3	84.7	65.2	
65	0.1	0.4	-0.4	0.9	0.6	0.3	0.9	0.4	0.4	0.5	0.3	0.7	1.1	27.1	38.3	46.9	56.6	40.2	
66	0.6	0.2	0.8	3.6	9.7	15.0	16.7	28.3	35.7	50.9	56.8	56.5	59.4						
67	0.7	0.6	0.4	0.6	0.8	0.5	1.2	1.2	2.2	2.6	2.4	2.9	5.6	51.9	59.6				
68	0.9	0.6	1.2	8.7	22.0	31.1	30.8	18.3	34.1										
69	0.5	0.8	1.0	1.0	1.5	5.5	8.9	14.5	18.7	19.4	20.9	22.9	21.6	60.8	77.2	78.9	76.3	81.0	72.0
70	0.3	0.7	0.3	0.0	0.6	0.9	4.0	6.9	9.4	12.7	12.3	14.3	17.6	70.1	91.8	92.8	83.1	84.7	88.0
71	0.5	0.8	2.7	5.9	15.8	22.8	32.2	43.3	46.3										
72	0.2	0.3	0.4	1.5	3.8	6.8	12.8	20.1	30.5	32.1	30.3	29.9	32.0	38.5	48.7				
73	0.4	0.5	0.9	2.8	9.7	20.5	27.5	34.7	34.1	35.1	32.7	31.5	35.3	36.9	36.3	40.9	40.6	42.1	39.0
74	0.3	1.0	1.2	3.4	8.6	15.4	20.6	29.3	30.6	30.6	34.3	48.4	60.5	71.0	68.4				

Groups of pigs (*n* = 12–39) were infected oronasally with 2 × 10^5^ TCID_50_ ASFV Estonia 2014 or 1 × 10^5^ TCID_50_ ASFV OURT/88/3. The ASFV OURT/88/3 group was re-infected with 1 × 10^6^ TCID_50_ ASFV OURT88/3 on 14 dpi. On the indicated dpi, the serum samples from the pigs were assessed for ASFV antibodies using INgezim PPA COMPAC (**A**,**C**) and I.D. Screen ASF ELISA (**B**,**D**) kits in ASFV Estonia 2014 (**A**,**B**) and ASFV OURT/88/3 (**C**,**D**)—infected groups of pigs. Cut-off value for INgezim PPA COMPAC: <40%, negative (white); 40–50%, doubtful (brown); ≥50%, positive (red). Cut-off value for I.D. Screen: <30%, negative (white); 30–40%, doubtful (brown); ≥40%, positive (red). The numbers in the tables are the % inhibitions for the INgezim PPA COMPAC kit and S/P% ratios for the I.D. Screen ASF Indirect ELISA Kit. Values presented are from 5 and 6 dpi for ASFV Estonia 2014 and ASFV OURT/88/3-infected groups of pigs, respectively.

**Table 3:** Side-by-side anti-ASFV antibody levels in the serum and meat exudate from pigs infected with ASFV OURT/88/3.

ASFV OURT/88/3 Final DPI: ID Screen ELISA Comparison for Serum and Meat Exudate Samples												
	P63	P64	P65	P66	P67	P68	P69	P70	P71	P72	P73	P74
**Serum**	42.8	65.2	40.2	59.4	59.6	34.1	72.0	88.0	46.3	48.7	39.0	68.4
**Diaphragm**	63.2	74.9	63.1	53.5	62.0	48.5	83.8	77.4	51.1	29.9	31.3	68.1
**Tongue**	52.8	72.7	75.8	60.0								
**Masseter (L)**	53.5	79.0	63.2	61.0	57.7	38.5	81.9	86.6	51.8	30.9	28.9	70.2
**Masseter (R)**	53.1	80.7	57.5	61.8	59.6	42.0	73.5	80.3	53.6	30.0	24.1	69.7
**Triceps (L)**	40.9	72.3	51.9	52.6								
**Triceps (R)**	41.4	96.6	54.2	49.6								
**Biceps (L)**	41.4	86.4	48.3	53.5								
**Biceps (R)**	31.3	82.0	43.8	62.4								

Groups of pigs (*n* = 12) were infected oronasally with 1 × 10^5^ TCID_50_ of ASFV OURT/88/3 and re-infected with 1 × 10^6^ TCID_50_ ASFV OURT88/3 on 14 dpi. At the end of the experiment, the serum and meat exudate were assessed for anti-ASFV antibodies using the I.D. Screen ASF ELISA kit and the results are presented together. Cut-off value for ID Screen: <30%, negative (white); 30–40%, doubtful (brown); ≥40%, positive (red).

## Data Availability

All data related to this study will be made available upon request.
